# Supplementary material for: Adapting cognitive diagnosis computerized adaptive testing item selection rules to traditional item response theory
Source: PLoS One. 2020 Jan 10;15(1):e0227196. doi: 10.1371/journal.pone.0227196 (PMC6953845; doi:10.1371/journal.pone.0227196)
Supplement: Attachment — Submitted filename: Response.docx [file pone.0227196.s001.docx]

**RESPONSE TO REVIEWERS: MANUSCRIPT ID PONE-D-19-15008**

October, 14, 2019

Dear Dr. Timo Gnambs:

Allow us to start by thanking you for the opportunity to revise and resubmit our manuscript “Exchanging Item Selection Rules from Cognitive Diagnosis Modeling to Traditional Item Response Theory in Computerized Adaptive Testing” (MANUSCRIPT ID PONE-D-19-15008). We would also like to thank you and the three reviewers for your constructive comments.

In the pages that follow, we respond to the comments brought up by the three reviewers. In our response, we specify the line numbers where the modifications have been made (manuscript file), and highlight those modifications in the revised manuscript with track changes file. In addition, we have checked the journal requirements described in the decision letter concerning the PLOS ONE style, data accessibility, and the funding statement. The code used in the simulation can be access at the following link: https://osf.io/eu4q6/. Regarding the Funding Statement, there was not additional funding received for this study. We provided in the title page an amended statement that declares all the support came from Grant PSI2017-85022-P (Ministerio de Ciencia, Innovación y Universidades, Spain).

We hope that you will find the revised version of our manuscript suitable for publication in PLOS ONE. We look forward to hearing from you regarding our submission.

Sincerely yours,

On behalf of the authors,

**A.	REVISION COMMENTS:**

**REVIEWER 1:**

**Overall, I have concerns about the merit of the study—from both the practical and theoretical viewpoints. The authors advocate the use of the double Kullback-Leibler divergence (DKL) and the discrimination index (GDI) as item selection methods in computerized adaptive testing (CAT); however, the overall performances of these measures are somewhat worrisome and I’m not sure if they provide any benefits over the existing methods. Figure 3 shows that the four item selection methods performed very comparably when EAP estimator was applied in estimation of the trait parameters (and thus no clear benefit). When ML estimator was used, DKL performed outperformed marginally after the administration of four items and yet showed the worst performance in the beginning stage of CAT. The authors argued for DKL because simple KL-based item selection can be disturbed by unstable trait estimates (p. 6 linear 140-143). The DKL however seemed to show even more disturbing item selection in the early stage of CAT. The item overlap results also suggest that DKL and GDI tended to suffer from skewed item usage. Overall, it was unclear if the suggested procedures present clear advantages despite the poorer performance in item exposure control and heavier computational load.**

Thank you for noticing this aspect. We believe it is important to distinguish between practical and theoretical merits of this study. From a theoretical point of view, we have presented two new ISRs (new in the context of IRT-CATs). Both of them have previously shown promising results in the context of CD-CATs, and both of them share an important characteristic: they do not rely on a single estimated trait level for the item selection. This implies that it can be theoretically expected that both will show a better performance in terms of accuracy. Given that, with a more peaked likelihood, the different ISRs tend to converge to PFI; we also expect those differences to be reduced as the number of administered items increase. So, from our point of view, our proposal has theoretical merit.

With respect to the practical merits, we agree that the plot in Figure 3 might lead to the impression that all the methods perform very similarly when the trait estimation method is EAP, probably because of the range of the RMSE values. With this plot we only aimed to provide a representation of the trends. For a thorough comparison the reader is referred to Table 1, where the results for *J* = 10 are provided. Please note that DKL always led to the lowest RMSE (with the only exception of *θ* = 2)*.* The row mean for DKL is .44, whereas the row means for PFI, KL, and GDI are .46, .47, and .47, respectively. The same is true for the bias. The differences can be considerably large. For example, average bias for DKL is .02 whereas the average bias for KL is .05. Differences are generally larger for extreme values of the trait level. For example, for *θ* = −2 the RMSE associated to DKL is .49 which is noticeably lower than the one associated with, for example, KL which is .56. We tried to make this point clearer in the manuscript by adding the row means in Table 1. We agree that the advantages of the new ISRs are more limited for longer CAT lengths, and we included the results for *J* = 20 in Table 1 to better illustrate the pattern. It can be observed that all ISRs mostly converged as the CAT progressed, and this was to be expected (lines 245-246). DKL attempts to deal with the problem of having a noisy point estimate of the trait level at the beginning of the test. For *J* = 20 the trait level will be reasonably well estimated, and then DKL and KL will provide similar results. Nonetheless, it is worth noting that differences, although small, were generally in favor of the proposed ISRs.

On the other hand, the latent trait level estimates at the very beginning of the test (e.g., 1-3 items) should not be interpreted. We aimed to provide the maximum amount of information in the figures, but surely the standard error for the trait level estimates is unacceptably large at that stage of the adaptive procedure, and this is true of any procedure. A tentative explanation for the DKL results with respect to this might be the use of very discriminating items at the very beginning of the test, as the likelihood or posterior distribution is not very peaked. This effect is more extreme for DKL than for KL, due to its double integral and the fact that it does not rely on a point estimate of the trait level. As shown by the overlap rate, the CAT with DKL is not very adaptive at the start of the test. Somehow this leads to lower accuracy. There are also other additional factors that prevent us from interpreting these results. First, all the IRSs, except for the PFI, used a weighting function (i.e., likelihood or posterior distribution). As we indicated in the Method (lines 270-274), two fictitious items were used to for the likelihood of the first item to be computed. These two items were not used after that, but differences in the performance of the ISRs at the very beginning of the test might be attributed to that. And second, Dodd’s method was used until the response pattern was not constant for the ML estimation, whereas the point estimate of the trait level is used in PFI and KL, which might partly explain the differences in the performance of the ISRs. In the revised version of the manuscript we warn the reader not to interpret the results for very short test lengths (lines 297-301).

With respect the test overlap, we agree with the reviewer that it can be a problematic issue in high-stakes situations. Please note, however, that for CATs longer than 13 items all the methods had overlap rates lower than .40. There is, nonetheless, a trade-off between security and accuracy. One possible way to reduce test overlap would be increasing item bank length or incorporating an item exposure control method. We provided a more in-depth discussion of this issue in the revised version of the manuscript (line 325 and 441-449).

With respect to computational load, we have included computation time as a dependent measure of our study. The proposed ISRs were slower but the computation time per item was always below a second, and the code can be further optimized, for example, by reducing the number of quadrature points. We describe and discuss these results in lines 370-374 and 399-403.

**The manuscript may be revised by lightening the strong advocacy of DKL and GDI (with the proper explanation of their pros and cons) and by elaborating more on the situations where the suggested methods can lend better utility.**

Thank you for this suggestion. We have included some text in the discussion section detailing the pros and cons of the DKL and GDI ISRs, and elaborating more on the situations where the new item selection rules can be more useful. We have tried to reduce the tone advocating in favor of our proposals (e.g., lines 32, 37-38, 325, 334-338, 394, and 398-399). DKL should be used whenever test security is not a major concern. In those conditions, although the benefit in terms of accuracy of this ISR can be small, we find no reason why accuracy should not be maximized, particularly when short tests are involved. However, in cases were test security is especially relevant, additional simulation studies are needed. As Barrada et al. [1] noted, comparing two different ISRs should be avoided when both their RMSEs and overlap rates differ at the same time. A fair comparison necessitates applying specific techniques to guarantee that the RMSEs or overlap rates of the ISRs are equalized, and this would require extensive manipulation of the maximum exposure rates. We included additional text describing this matter in lines 444-447.

**REVIEWER 2:**

**This paper proposed two new item selection methods for IRT-based CAT from two CD-CAT-based algorithms one of which demonstrate excellent performance in measurement efficiency and item exposure rates. After careful reading of this manuscript, I recommend its publication in Plosone with some minor revision.**

We appreciate your comments and suggestions to improve the manuscript.

**The main problem is that the authors did not explain the similarity/difference in the double KL (DKL) and the existing Bayesian KL in IRT-CAT. It makes more sense to use the Bayesian KL as a baseline method rather than the KL method.**

Perhaps we have not fully understood the reviewer's point here. In our simulation study we used the Bayesian KL [2]. What we, for simplicity, note as KL item selection rule is the Bayesian KL when EAP estimator is used; when ML estimator is used, it is KL weighted by likelihood.

The KL method that we used is described in Equation 4:

|  | $j=\mathrm{argmax}_{i\in B_{q}}\int_{-\infty}^{\infty} KL_{i}\left(\theta\left\Vert\hat{\theta}\right.\right)W\left(\theta\right)d\theta$, | (4) |
| --- | --- | --- |

$W(\theta)$was equal to the likelihood function when ML estimation was used and equal to the posterior distribution when EAP was used. The prior distribution of $\theta$(i.e., $f_{0}(\theta)$) was set to U[–4, 4]. As the prior was uniform, there were, in fact, no differences between $W(\theta)$by estimator.

We have tried to further explain the similarities/differences between the double KL (DKL) and the existing Bayesian KL. For doing so, we have expanded the limitations of the PFI method, in order to better describe the benefits of KL and DKL in lines 117-132 and 147-170. The main ideas are described in the following:

Fisher information is the discrimination power between two close $\theta$ values [2]. This implies two limitations for PFI. First, that the discrimination power between distant $\theta$ values is not considered. Second, that the selection rule implicitely assumes that the deviation of $\hat{\theta}_{q}$ from $\theta$ is small.

The advantage of KL is described as:

*KL, as a global measure of information, on the contrary, consider the information content in the item with respect to a broad range of latent trait levels, addressing the first limitation.*

But KL does not address the second limitation:

*KL performance is still dependent on the proximity between the estimated trait level and the real trait level.*

The rationality of why DKL should outperform KL is described in this paragraph:

*The advantage of this index is that it does not use the estimated latent trait at all (compare this equation with Equation 4). This is a nice feature because this estimate can be very noisy in the early stages of the CAT. The best way of dealing with this undesirable fact is to use an ISR that does not use these noisy estimates.*

We understand that this was not clear enough in the manuscript. We hope it is more understandable now.

**REVIEWER 3:**

**This work evaluates the performance of two item selection rules that were originally proposed in the Cognitive Diagnosis (CD)-Computerized Adaptive Testing (CAT) framework, the double Kullback-Leibler information (DKL) and the generalized deterministic inputs, noisy “and” gate model discrimination index (GDI), in the setting of traditional CAT under the unidimensional Item Response Theory (IRT) model. The authors gave a very nice review of the existing works and performed a simulation study to compare the DKL and GDI with two existing methods in CAT. Both the measurement accuracy and test security were compared, and the simulation shows promising results of the two proposed approaches (DKL and GDI). This is an interesting study and it is well written. As pointed out by the authors, it is one of the first times that CDM methodologies are applied to the traditional IRT context. I have the following specific comments, which I hope would be helpful.**

Thank you for your comments and the interesting reflections you propose.

**1. As the authors mentioned on Page 6, Chang and Ying also discussed the idea of using two integrals for KL considered in a range around \hat\theta. Would the authors comment on how this “locally” double-integral approach compares with the proposed globally integrals in DKL?**

Thank you for this suggestion. We have included information regarding this point (lines 147-166):

*This idea of using two integrals was already briefly mentioned in the discussion of* *Chang and Ying’ study (see equation 29), but not with KL weighted by posterior, but for KL considered in a range around* $\hat{\theta}_{q}$*. This ISR, which we will call double KL within intervals (DKLI) was defined as:*

|  | $j=\mathrm{argmax}_{i\in B_{q}}\int_{\hat{\theta}_{q}-\eta_{q}}^{\hat{\theta}_{q}+\eta_{q}} \int_{\hat{\theta}_{q}-\delta_{q}}^{\hat{\theta}_{q}+\delta_{q}} KL_{i}\left(\theta_{1}\left\Vert\theta_{2}\right.\right)d\theta_{1}d\theta_{2}$*,* | *(6)* |
| --- | --- | --- |

*where* $\eta_{q}$ *and* $\delta_{q}$ *determine the amplitude of the interval around* $\hat{\theta}_{q}$*. This amplitude decreases with each new administered item, as the uncertainty about the estimated trait level is supposed to be reduced as the test goes on.*

*In our proposed ISR –DKL– the next item to be selected is given by*

|  | $j=\mathrm{argmax}_{i\in B_{q}}\int\int_{-\infty}^{\infty} KL_{i}(\left.\theta_{1}\right\Vert\theta_{2})\mathrm{W}(\theta_{1})\mathrm{W}(\theta_{2})d\theta_{1}d\theta_{2}$*.* | *(7)* |
| --- | --- | --- |

*DKLI differs in three relevant points with respect to our proposed DKL. First, DKLI still needs the computation of an estimated trait level after each new administered item.* $\hat{\theta}_{q}$ *is still part of the equation. With DKL the estimated trait level is not used at all for item selection. Second, with DKLI all the computed KL values are equally weighted. With Bayesian KL or KL weighted by likelihood (equation 4) or with DKL, KL values are weighted by the best available evidence, that is, likelihood function or posterior distribution. Third, DKLI dismiss potentially relevant information, all the one outside the interval. With Bayesian KL and KL weighted by likelihood, all the possible pairs of values with respect to the estimated trait level are compared. For DKL, all the possible pairs of values are compared. Previous studies have shown that KL weighted by likelihood reduced measurement error when compared with KL based on (a single) interval* [3]*. We considered that there were solid reasons for not testing DKLI in this study.*

**2. The evaluation of DKL involves with double integration. Would the authors comment on the computation cost for this method?**

Thank you for pointing this out. We have included computation cost as a dependant measure of our study. We have included the following new sentences.

In the *Method* section (lines 293-294):

*Finally, the time required to select a single item for each ISR was recorded.*

In the *Results* section (lines 370-374):

*The code was run on a computer with processor of 3.0 GHz. The average times, in milliseconds, for selecting each item were 0.3 ms/item for PFI, 11.2 ms/item for KL, 3.9 ms/item for GDI, and 219.4 ms/item for DKL. As the computation time is dependant among many computer characteristics, we also computed the ratio, relative to PFI: KL was 36.5 times slower, 12.6 slower for GDI, and 713.3 times slower for DKL.*

In the *Discussion* section (lines 399-404):

*The proposed ISRs were slower than PFI, especially DKL. Acknowledging this, the computation time per item was always markedly below a second, so we deem this differences as trivial in any applied context. It should be noted that the simulation code was not written to optimize execution times. DKL, with its double integration, used 81 × 81 = 6561 quadrature points. We expect that this number could be reduced without any impact in accuracy.*

**3. I noticed from the simulation results that for the DKL method, there seem to have an increment of MSE for the first two items in Figure 3. Is this due to simulation error or is there any other reason for such trend?**

Thank you for pointing this out. The plots in Figs 1-6 were included to provide a representation of the trends. However, it is important to note that the results at the very start of the CAT (1-3 items) should be interpreted with caution. The reason is that the ISRs might be affected differently by some specific characteristics of the CAT algorithm at the beginning of the test (e.g., Dodd’s method, the use of two fictitious items in the likelihood computation for the first item). It is not due to simulation error. After the administration of a few items, the weighting function (i.e., likelihood or posterior distribution) and the point trait level estimates that are used in the computation of the different ISRs are more stable and less affected by the simulation design. This improves the trait level estimation. We understand this was not clear enough in the previous version of the manuscript. In the revised version of the manuscript we warn the reader not to interpret the results for very short test lengths (lines 297-301).

**4. The performance of the DKL seems worse than KL for the first few items. Is there any intuition why this would happen?**

We can offer a tentative explanation. At the beginning of the test, DKL selects overall very discriminating items, as the likelihood or posterior distribution is not very peaked. This effect is more extreme for DKL than for KL, due to its double integral and the fact that it does not rely on a point estimate of the trait level. As shown by the overlap rate, the CAT with DKL is not very adaptive when the test starts. Somehow this leads to lower accuracy. Additionally, as we note in the response to your previous comment, the weighting function that is used in the computation of DKL and GDI is initially approximated using two fictitious items. These two items are removed after the administration of the first item. Something similar occurs with KL. Until a non-constant pattern is used, KL uses an heuristic estimate of the trait level (i.e., Dodd’s method). These sudden changes might partly explain the strange pattern of results at the beginning of the test. Bearing this in mind, in the revised version of the manuscript we warn the reader not to interpret the results for very short test lengths (lines 297-301).

**Related to this question, would the performance be further improved if selecting the first few items with KL and then using DKL for later items?**

Although there have been several proposals to switch between ISR –mainly for the improvement of test security; e.g., [4–6]–, in this point we have followed the common practice in CATs: to maintain the same ISR all through the test. We consider that this is the best option for the present study: before combining ISRs we needed an in-depth evaluation of the independent performance.

**5. As the DKL and GDI were originally proposed for CDMs with multiple attributes, I was wondering if these methods can also be extended to multidimentional IRT models? A discussion would be helpful.**

There exists a prior work by Wang, Chang, and Boughton [7] where the authors extent the KL index to the multidimensional case. Their approximation considers the point estimates for the *k* latent trait levels. DKL and GDI do not consider the point estimates. It would be possible to apply a similar logic in the multidimensional scenario. This can be, however, computationally expensive since it would require integrating across multiple dimensions. We included a discussion on this topic in lines 455-459.

**B.	REFERENCES:**

1. Barrada JR, Olea J, Ponsoda V, Abad FJ. A method for the comparison of item selection rules in computerized adaptive testing. Applied Psychological Measurement. 2010;34: 438–452. doi:10.1177/0146621610370152

2. Chang H-H, Ying Z. A global information approach to computerized adaptive testing. Applied Psychological Measurement. 1996;20: 213–229. doi:10.1177/014662169602000303

3. Barrada JR, Olea J, Ponsoda V, Abad FJ. Item selection rules in computerized adaptive testing: Accuracy and security. Methodology. 2009;5: 7–17. doi:10.1027/1614-2241.5.1.7

4. Barrada JR, Abad FJ, Olea J. Varying the valuating function and the presentable bank in computerized adaptive testing. Span j psychol. 2011;14: 500–508. doi:10.5209/rev_SJOP.2011.v14.n1.45

5. Li YH, Schafer WD. Increasing the homogeneity of CAT’s item-exposure rates by minimizing or maximizing varied target functions while assembling shadow tests. J Educational Measurement. 2005;42: 245–269. doi:10.1111/j.1745-3984.2005.00013.x

6. Leung C-K, Chang H-H, Hau K-T. Computerized adaptive testing: A mixture item selection approach for constrained situations. British Journal of Mathematical and Statistical Psychology. 2005;58: 239–257. doi:10.1348/000711005X62945

7. Chung W, Chang H-H, Boughton KA. Kullback-Leibler information and its applications in multi-dimensional adaptive testing. Psychometrika. 2011;76: 13–39. doi: 10.1007/S11336-010-9186-0
